# Health behaviour changes in partners of women with recent gestational diabetes: a phase IIa trial

**DOI:** 10.1186/s12889-018-5490-x

**Published:** 2018-05-02

**Authors:** Anne-Sophie Brazeau, Sara J. Meltzer, Romina Pace, Natasha Garfield, Ariane Godbout, Leslie Meissner, Elham Rahme, Deborah Da Costa, Kaberi Dasgupta

**Affiliations:** 1 School of Human Nutrition, McGill University, Montréal, Canada; 2 Department of Medicine, McGill University, Montréal, Québec Canada; 3 Division of Endocrinology, Centre de recherche du Centre Hospitalier de l’Université de Montréal, Montréal, Canada; 4 St. Mary’s Hospital Centre, Montréal, Canada

**Keywords:** Gestational diabetes, Type 2 diabetes, Prevention, Dietary behaviors, Exercise, Couples

## Abstract

**Background:**

We recently demonstrated that a gestational diabetes history in mothers is associated with higher postpartum incident diabetes not only in mothers but also in fathers. In the present study, we examined changes in health behaviours and cardiometabolic profiles in both mothers and partners who participated in a diabetes prevention program within 5 years of a gestational diabetes pregnancy.

**Methods:**

Couples were enrolled into a 13-week program that included 5 half-day group sessions and web/telephone-based support between sessions. It was designed in consultation with patients and previously studied in mothers. We computed mean changes from baseline (95% CI) for physical activity, eating, and sleep measures, and cardiometabolic parameters (fasting and 2-h post glucose load plasma glucose, BMI, blood pressure) in both partners and mothers.

**Results:**

Among 59 couples enrolled, 45 partners (76%) and 47 mothers (80%) completed final evaluations. Baseline cardiometabolic measures averaged within normal limits. Similar to mothers, partners increased physical activity (+ 1645 steps/day, 95%CI 730, 2561; accelerometer assessed moderate-to-vigorous physical activity + 36.4 min/week, 95% CI 1.4, 71.4) and sleep duration (+ 0.5 h/night, 95% CI 0.1, 0.9) and reduced the sodium-to-potassium ratio of food intake (− 0.09 95% CI -0.19, − 0.001). No conclusive changes were observed in glucose measures or insulin resistance; in analyses combining mothers and partners, systolic blood pressure decreased (− 2.7 mmHg, 95% CI -4.4, − 1.0).

**Conclusions:**

Partners and mothers demonstrated improved physical activity, sleep, and dietary quality. Baseline cardiometabolic profiles averaged at normal values and there were no changes in glucose or insulin resistance; some blood pressure impact was observed. While strategies need to be developed to attract participants at higher cardiometabolic risk, this study demonstrates that partners of women within 5 years of a gestational diabetes diagnosis can be recruited and do achieve health behaviour change.

**Trial registration:**

ClinicalTrials.gov: NCT02343354 (date of registration: January 22, 2015).

**Electronic supplementary material:**

The online version of this article (10.1186/s12889-018-5490-x) contains supplementary material, which is available to authorized users.

## Background

Gestational diabetes (GDM) is a well-established postpartum diabetes risk indicator. A systematic review indicates that GDM confers a 7-fold risk increase [1], with 30–70% of women developing type 2 diabetes within 10 years of a GDM pregnancy [2]. GDM is also associated with postpartum development of metabolic syndrome [3], hypertension and cardiovascular disease [4]. The American Diabetes Prevention Program trial demonstrated that an intervention strategy focusing on eating and physical activity habits can markedly reduce diabetes risk following GDM [5], although attracting younger mothers is challenging. Studies to date have tailored interventions to mothers, but mothers themselves express a need for partner collaboration for health behaviour change [6].

Collaboration by partners for diabetes prevention may not only be important for the mother’s health but also for the partner’s health. In a previous study, we demonstrated that GDM in mothers is associated with incident diabetes in fathers [7]. Shared diabetes risk in partners is likely mediated by shared partner behaviours, as identified through analyses of the English Longitudinal Study of Ageing [8] and the Framingham Heart Study [9]. Shared risk and shared behaviours arguably constitute levers for collaboration between partners for diabetes prevention.

We have been developing a multimodal health behaviour change strategy following GDM, and testing it iteratively through a pre intervention/post intervention change design (The MoMM program: M*indful m*O*vement,* M*indful eating,* Mi*ndful living*). The aim is to optimize recruitment and engagement through these steps to ultimately launch a large randomized controlled trial. The key elements of this intervention are group sessions with preparation of healthy meals under a dietitian’s supervision and walks, games, and exercises with an exercise physiologist. Both components are coupled with discussion of how to realistically optimize eating and physical activity patterns at home. Sessions are held once per month over 4 to 6 months. On-site childcare is available. We previously reported effects in mothers [10]. The present study enrolled and evaluated both mothers and their partners (MoMM-ii). The aim of this study was to adapt the previous program to more comprehensively include partners and to evaluate changes in health behaviors and cardiometabolic profiles in both mothers and partners following participation in the MoMM-ii program.

## Methods

### Ethics, consent and permissions

This was a single-arm interventional study (ClinicalTrials.gov: NCT02343354) examining pre- to post intervention changes among mothers within 5 years of a GDM pregnancy and their partners. The protocol was approved by McGill University’s Faculty of Medicine Institutional Review Board and all participating institutions (McGill University Health Centre, Centre Hospitalier de l’Université de Montréal, Sir Mortimer General Jewish General Hospital, St-Mary’s Hospital and Concordia University). Participants provided written informed consent.

### Eligibility criteria and recruitment

**Fig. 1:**
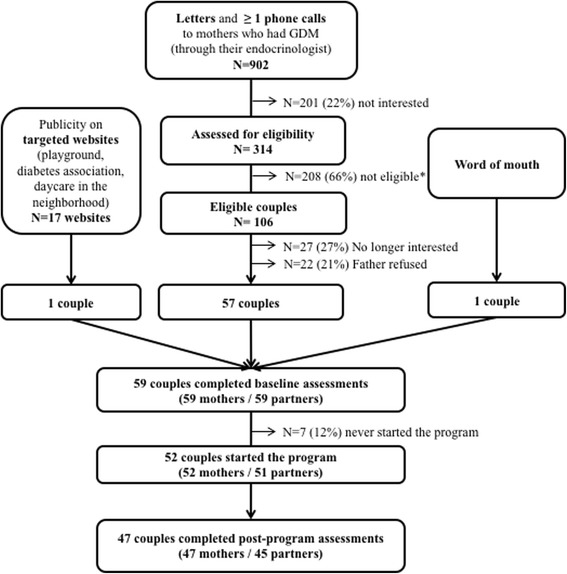
Participants’ Flow chart. *Not eligible: no GDM at last pregnancy = 13; GDM > 5 years ago = 2; no partner = 15; diagnosis of diabetes in one couple member = 29; not speaking French or English = 28; not available on weekends = 63; smokers = 26; currently pregnant = 22; trying to conceive = 10

Mothers were required to have had GDM pregnancy within the prior 5 years and a partner who was willing to enrol. Same-sex partners were not excluded. Exclusion criteria were having diabetes (any type), current use of antihyperglycemic medication, pregnancy or attempting to become pregnant, current smoking, and/or co-morbid conditions or medications (e.g., antipsychotic drugs or steroid hormones) that could impact weight or ability to engage in moderate intensity physical activity. Recruitment was primarily through GDM clinics (McGill University Health Centre, Centre Hospitalier de l’Université de Montréal, Sir Mortimer General Jewish General Hospital, St-Mary’s Hospital; referrals by clinic staff; Fig. 1). Potential participants, referred by clinic staff, received a letter and a phone call (by ASB or one of the research assistants) inviting them to contact the study team for further information about the program. The study was also publicized through websites (e.g., daycares, diabetes associations).

### Intervention

**Fig. 2:**
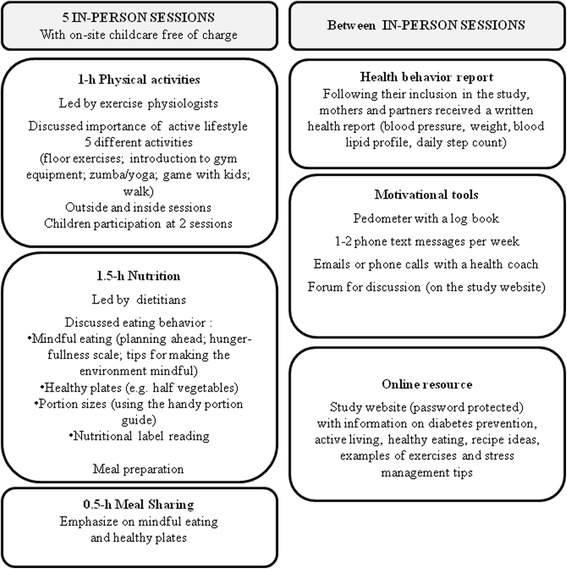
Key components of the MoMM-ii program

Participants were invited to 5 three-hour group-based sessions at 3-week intervals over 13 weeks (Fig. 2). Sessions were held at Concordia University’s PERFORM centre, a research and teaching facility equipped with a kitchen (four work stations) and an exercise area. During the first hour of each session, under an exercise physiologist’s supervision, they performed floor exercises, developed familiarity with exercise equipment, participated in group games, and learned how to use a step counter to progressively achieve ≥10,000 steps/day [11]. Over the following 2 h, a registered dietitian discussed eating behaviour and nutrition and supervised participants in the preparation of a balanced meal. Between sessions, participants had access to a study-specific password protected website (e.g., discussion forum, recipes, exercises to do at home). One to two phone text messages were sent each week with tips to optimize health behaviours.

### Assessments

Demographic information, family history, and past medical history were queried. Postal code information was used to derive the Institut National de Santé Publique du Québec material deprivation index, a quintile ranking derived from census dissemination area level scores based on proportion without high school diploma, employment/population ratio, and average income [12]. The assessments described below were performed both at baseline and following the intervention. Assessments were at the PERFORM Centre (Concordia University). ASB, RP, and KD conducted the assessments with assistance from two research assistants and two students.

#### Cardiometabolic

*Anthropometric measures*. Weight to the nearest 0.1 kg (Digital Physician Scales, model 140–10-6, Rice Lake Weighing Systems; light clothing, shoes removed) and height to the nearest 0.1 cm (Stadiometer PE-WM-60-84-BRG2, Perspective Enterprises) were assessed and body mass index (BMI) calculated. *Oral glucose tolerance test and insulin resistance.* Plasma glucose (glucose oxidase method) and insulin (ELISA method) were measured on blood samples drawn in the fasting state and 120 min following ingestion of a 75 g glucose drink. The Homeostatic Model Assessment Insulin resistance (HOMA-IR) [13] and insulin sensitivity index (ISI_0,120_) [14] were computed. *Other serum markers.* Lipid parameters (Total-cholesterol, HDL-chol, Triglycerides) were measured on fasting blood samples (Piccolo xpress technic) and LDL-chol was calculated. *Blood pressure*. Blood pressure was assessed in a seated position with the arm supported following a 5-min rest period. Six measurements at 1-min intervals were recorded (Mindray Accutor –V Vital Signs Monitor). The last 5 systolic (SBP) and diastolic blood pressure (DBP) measurements were separately averaged.

#### Behavioural measures

*Dietary intake and eating behaviour*. Food consumption was assessed through a semi quantitative, self-administered, validated Food Frequency Questionnaire [15]. To be included in the analysis, participants needed to report plausible intake based on their total energy estimate adapted for breastfeeding status and weight [16]. Data are presented as number of daily serving of each food groups and in percentage of total energy for macro-nutrients. Fiber intakes are presented as gram per 1000 kcal. Intakes of sodium and potassium are presented in absolute values and as a ratio (sodium-to-potassium ratio). The joint effect of sodium and potassium is associated with hypertension and cardiovascular events [17]. Additional questions included the frequency of eating prepared convenience foods or restaurant meals [18], skipping breakfast, and eating in front of the television; self-perceived ability to cook from basic ingredients was also rated (7-point scale) [19]. *Physical activity-sedentary behaviour.* Daily step counts were measured using Piezo SC-Step X Health System pedometers over 7 days [20]. Participants also wore a multi-axial accelerometer (ActiGraph GT3X, Actigraph LLC., Pensacola, FL). Accelerometer data analyses required at least 10 h per day for a minimum of 4 days. Other questionnaires included the Physical Activity and Sedentary Behaviour Questionnaire (PASB-Q) of the Canadian Society for Exercise Physiology, the Occupational Sitting And Physical Activity Questionnaire (OSPAQ) [21] and questions that queries readiness to perform more physical activity (i.e., stages of change) [22].

#### Patient oriented outcomes

Self-administered questionnaires included the Weight Efficacy Lifestyle (WEL; scores 1 to 9) [23] which assesses eating-related self-control and the Mindful Eating Questionnaire (MEQ; scores 1 to 4) [24]. The Pittsburgh Sleep Quality Index (PSQI) was also administered [25] given the improvements in sleep that may be associated with higher physical activity levels. The PSQI assesses sleep quality over the previous month with a global score ≥ 6 differentiating poor sleepers and good sleepers [25]. At the last visit, participants were invited to share their impressions of the program (yes/no and open-ended questions).

### Statistical analysis

Descriptive statistics are presented as means and standard deviations (SD), median or proportions, as appropriate. Mean changes with 95% confidence intervals were calculated for normally distributed data. Wilcoxon rank sum tests were used if departure from normality was significant; Exact McNemar’s tests for proportion were used to compare changes from pre-to-post program. We evaluated these metrics for mothers and partners combined and separately.

## Results

### Recruitment

Recruitment occurred over 3 months, largely through collaborating clinics. A total of 902 individuals received an invitation letter and a phone call (Fig. 1). Among those who demonstrated initial interest, approximately one quarter was no longer interested when invited to enrol and one fifth was not eligible because the partner did not wish to participate. In terms of socioeconomic differences between participants and non-participants, while those from the most deprived neighbourhoods did participate, a lower proportion of participants resided in the two most deprived neighbourhood quintiles (i.e., one third of participants vs. half of non-participants). Among the 118 individuals who completed baseline evaluations, 15 (13%; 8 partners and 7 mothers) did not attend any sessions.

### Baseline characteristics

The 59 couples enrolled were from a variety of ethnocultural backgrounds (Table 1). Two were same-sex couples and the remainder were mother-father pairs. On average, couples had been together for approximately a decade and had 2 children. Approximately 70% of both mothers and partners had completed a university degree and over half were in the highest household income category (i.e., > $80,000 CAD).

**Table 1:** Participants’ baseline characteristics

	Partners	Mothers
	*N* = 59	*N* = 59
Age, yr. (mean; sd)	40.8 SD 4.5	37.8 SD 4.6
Ethnocultural background, n (%)		
White	24 (40.7%)	20 (33.9%)
West Asian	15 (25.4%)	17 (28.8%)
Asian	9 (15.3%)	10 (16.9%)
Black	9 (15.3%)	10 (16.9%)
Latin American	2 (3.4%)	2 (3.4%)
Having completed a University degree; n (%)	41 (69.5%)	43 (72.9%)
Household income; n (%)		
< 30,000 CAD	6 (10.2%)	
30,000–80,000 CAD	22 (37.3%)	
> 80,000 CAD	31 (52.5%)	
Couple’s duration, yr. (mean; sd)	10.8 SD 5.0	
Children, number (mean; sd)	1.9 SD 0.9	
Time since last pregnancy, yr. (mean; sd)	1.9 SD 1.1	

Approximately three quarters of partners and more than half of mothers had a BMI ≥ 25 kg/m^2^ at baseline. Partners averaged just above the low active step count threshold (7500 steps/day) and mothers’ activity levels were lower. Eating behaviors were generally good. Specifically, most did not skip breakfast. Roughly half ate out fewer than three times/month and reported never eating in front of the television. (71% partners; 79% mothers) and half ate out fewer than 3 times/month (46% partners; 59% mothers) and more than half reported never eating in front of the television (57% partners; 55% mothers). A high proportion reported good cooking skills before study entry (61% of partners and 88% of mothers). Partners averaged close to the optimal 7 to 8 h of sleep while mothers were slightly below this level [26]. Mean values for cardiometabolic measures were within normal limits (i.e., glucose, blood pressure, lipid levels, insulin resistance and sensitivity; Table 2). Five partners (8%) and nine mothers (15%) had prediabetes (i.e., impaired fasting glucose: fasting glucose ≥6.1 mmol/L; and/or impaired glucose tolerance: 2-h post load value ≥7.8 mmol/L).

**Table 2:** Cardiometabolic measures

	All participants		Partners		Mothers	
	Before; mean (sd)	Mean change [95% CI]	Before; mean (sd)	Mean change [95% CI]	Before; mean (sd)	Mean change [95% CI]
Weight, kg	75.6 (14.9)	-0.1 [−0.5, 0.3]	81.7 (14.7)	−0.27 [− 0.87, 0.34]	70.1 (12.8)	− 0.04 [− 0.59, 0.52]
BMI, kg/m^2^	26.8 (4.3)	− 0.05 [− 0.19, 0.09]	27.1 (4.0)	−0.09 [−0.29, 0.11]	26.5 (4.6)	−0.01 [− 0.22, 0.20]
Steps, steps/d	7481 (2314)	1355 [740, 1970]	7553 (2871)	1645 [730, 2561]	7410 (1606)	1065 [215, 1915]
Systolic Blood pressure, mmHg	119.2 (13.3)	−2.7 [−4.4, −1.0]	123.7 (13.3)	−1.9 [−4.3, 0.5]	115.1 (12.1)	−3.4 [− 5.8, − 1.1]
Diastolic Blood pressure, mmHg	72.6 (9.7)	−1.8 [−3.3, −0.4]	75.6 (10.0)	−2.5 [− 4.7, 0.4]	69.9 (8.6)	−1.2 [− 3.1, 0.7]
Fasting plasma glucose, mmol/L	5.2 (0.5)	0.08 [0.01, 0.14]	5.2 (0.5)	0.09 [0.01, 0.19]	5.2 (0.6)	0.07 [−0.01, 0.14]
2-h plasma glucose, mmol/L	5.5 (1.6)	0.15 [−0.19, 0.48]	5.1 (1.5)	0.20 [−0.28, 0.69]	5.8 (1.6)	0.16 [−0.33, 0.66]
Fasting insulin, μU/mL	4.9 (3.1)	0.9 [0.3, 1.6]	5.0 (3.7)	0.9 [−0.2, 1.9]	5.0 (2.6)	0.9 [0.1, 1.1]
2-h insulin, μU/mL	30.2 (31.3)	6.8 [−0.7, 14.2]	31.5 (38.8)	7.0 [−6.7, 20.7]	28.5 (21.6)	19.4 [−7.3, 46.1]
HOMA-IR	1.2 (0.9)	0.21 [0.04, 0.38]	1.1 (1.2)	0.21 [−0.08, 0.49]	1.2 (0.6)	0.23 [0.04, 0.42]
ISI _0,120_mg × L^2^/mmol×μU × min	94.0 (38.5)	−6.2 [−12.9, 0.5]	104.4 (39.9)	−6.5 [−17.3, 4.2]	83.5 (34.4)	−5.9 [−14.3, 2.5]
Chol-tot, mmol/L	4.6 (0.7)	−0.13 [−0.24, −0.02]	4.6 (0.7)	−0.12 [−0.30, 0.06]	4.7 (0.7)	−0.13 [−0.27, 0.01]
HDL-chol, mmol/L	1.3 (0.3)	−0.02 [− 0.06, 0.02]	1.2 (0.3)	−0.01 [−0.07, 0.04]	1.4 (0.3)	−0.02 [−0.08, 0.04]
Triglycerides, mmol/L	1.2 (0.7)	−0.04 [−0.14, 0.06]	1.2 (0.7)	−0.07 [−0.22, 0.09]	1.1 (0.7)	−0.01 [−0.15, 0.13]
LDL-chol (calc), mmol/L	2.8 (0.6)	−0.06 [−0.15, 0.03]	2.8 (0.7)	−0.00 [−0.13, 0.13]	2.7 (0.6)	−0.12 [−0.24, 0.00]

One participant was excluded for glucose and insulin values because of a recent stop of blood glucose lowering medication for diabetes prevention. Two participants refused to do the OGTT at post-program assessment and only fasting values were available. Data includes one same sex couple

*BMI* body mass index, *HOMA-IR* Homeostasis model-assessment-estimated insulin resistance, *ISI*_*0,120*_ insulin sensitivity index

### Session attendance

Nearly 90% of participants attended at least one session (86% of partners and 88% of mothers). Among them, 64% of partners and 75% of mothers attended at least 3 of the 5 scheduled sessions. Overall, among the 59 couples who completed baseline evaluations, 45 partners (76%) and 47 mothers (78%) completed the post-intervention assessment. The changes presented are from those participants.

### Changes

**Fig. 3:**
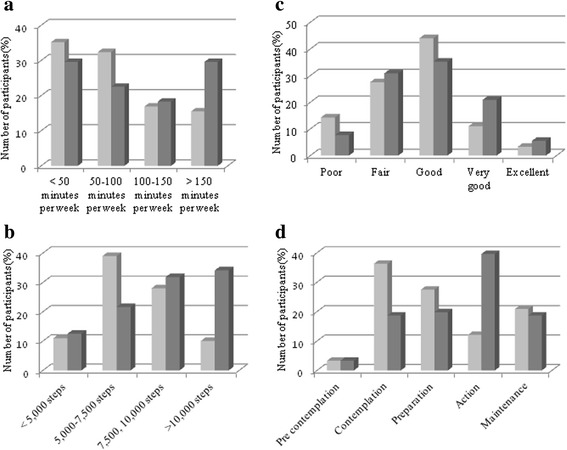
Change in physical activity behaviors. Light grey = before the program; Dark grey = after the program. **a** Moderate-to-vigorous physical activity. **b** Daily step counts. **c** Perception of physical fitness. **d** Stage of change for readiness to be physically active. Figure 3a includes all 71 participants with pre and post data. (Wilcoxon signed rank test, *p* = 0.007). Figure 3b includes all 88 participants with pre and post data available (Wilcoxon signed rank test, *p* < 0.001). Figure 3c (Wilcoxon signed rank test, *p* = 0.017) and Fig. 3d (Wilcoxon signed rank test, *p* = 0.002) include all 90 participants with and post data available

There were improvements in step counts (average change 1355 steps/day, 95% CI 740, 1970; partners: 1645 steps/day, 95% CI 730, 2561; mothers: 1065 steps/day, 95% CI 215, 1915) and MVPA (average change 27.3 min/week 95% CI 4.9, 49.0; partners: 36.4 min/week, 95% CI 1.4, 71.4; mothers: 18.0 min/week, 95% CI -10.2, 46.2; Fig. 3; Additional file 1: Table S2). Partners had a conclusive 1-h reduction in self-reported daily sitting time (Additional file 1: Table S2) with a similar trend in mothers.

**Fig. 4:**
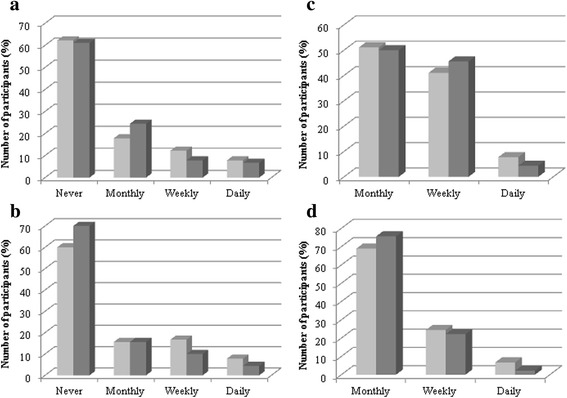
Change in eating behaviors. Light grey = before the program; Dark grey = after the program. **a** Eating in front of the television. **b** Eating out. **c** Skipping breakfast. **d** Eating convenience meal. Based on 90 participants with pre and post program data. Wilcoxon signed rank, Fig. 4a *p* = 0.445; Fig. 4b *p* = 0.674; Fig. 4c *p* = 0.004; Fig. 4d *p* = 0.106

By the end of the program, one quarter to one fifth skipped breakfast less frequently (Fig. 4). One third of partners (31%) and one fifth of mothers (20%) reported improved cooking ability (26% overall; Wilcoxon signed rank test *p* = 0.029). Total daily energy intake and macronutrient distribution were similar before and after the program. There was trend towards reduced intake of grain products and increased in vegetable and fruit consumption (Additional file 1: Table S1). This likely accounted for the small but conclusive reduction in sodium-to-potassium ratio observed.

Sleep duration increased by an average of half an hour per night (0.5 h, 95% CI 0.1, 0.9; baseline 6.9 h, SD 1.3) in partners and by over an hour in mothers (1.3 h, 95% CI 0.8, 1.6; baseline 6.4 h/night, SD 1.3). The proportion reporting poor sleep quality declined by greater than 10% overall (from 42% to 31%; exact McNemar’s test, *p* = 0.041; partners 9% reduction; mothers 13%). Systolic blood pressure decreased overall in a combined analysis of partner and mother data -and in mothers alone (Table 2). No improvements in plasma glucose, lipid profile or BMI were observed.

### Participant perceptions of the program

Participants perceived sessions as useful in promoting healthy eating (93% partners; 90% mothers) and the incorporation of ‘hands-on’ cooking was deemed to be an important component to this end (89% partners; 88% mothers). Physical activity education sessions were viewed as helpful in increasing activity levels (74% partners; 88% mothers) and a large proportion indicated that the pedometer was a valuable tool (92% partners; 98% mothers). Many noted that the availability of on-site childcare facilitated session attendance (89% partners; 90% mothers). However, less than half (41% of partners; 48% of mothers) indicated that the MoMM-ii website was useful to them, about one fifth (18% of partners; 21% of partners) used the suggested phone applications, and none used the website discussion forum.

## Discussion

This phase IIa trial in couples within 5 years of a GDM history demonstrates that a group based multimodal program (meal preparation, physical activity, on-site childcare, discussion, between-session contact) can engage not only mothers but also partners, and can help both achieve favourable changes in physical activity, dietary choices, and sleep measures. Among the 87% of participants who attended at least one session, three quarters of mothers and 64% of partners completed at least 3 of the 5 scheduled sessions. At baseline, physical activity and sleep duration were under recommended levels; both mothers and partners demonstrated clinically important increases in objective measures of step counts and moderated to vigorous physical activity and increases in sleep duration and quality. Baseline eating behaviours were good at baseline but there were nonetheless some favourable changes including a reduction in sodium-to-potassium intake ratio. However, similar to post GDM programs targeting mothers alone [27], in this program recruitment of those at risk continues to be challenging. Baseline cardiometabolic measures were well within normal limits on average and eating behaviours were generally good. Consequently, ability to demonstrate impact on BMI and cardiometabolic profiles was limited.

Partners experienced conclusive increases in physical activity as assessed by objective measurement of both step counts (additional 1645 steps/day) and MVPA (additional 36 min/week), and this was corroborated through self-reported measures. Similar effects were observed in mothers. Pedometer-based programs that focus exclusively on step count changes demonstrated increases of 2000 steps/day [28]. The step count increase level that we observed (1355 steps/day) was notable particularly given that the focus of the program was not strictly on steps. Perhaps even more importantly, we observed an objectively-assessed 30-min increase in MVPA, leading to a greater proportion of participants reaching the 150 min/week of MVPA. This may reflect the inclusion of a variety of physical activity options other than walking alone. Increasing MVPA improves cardiorespiratory fitness, reduces symptoms of depression and may help to reduce heart disease, stroke, high blood pressure and diabetes [29].

We also observed a trend towards increased consumption in vegetables and fruit as well as a reduction in grain intake, consistent with a reduction in the sodium-to-potassium intake ratio [30]. The effects on eating behaviours and dietary intake were less pronounced than effects on physical activity and there was no change in BMI. This is likely in part due to the fact that eating behaviours and dietary intakes were more adequate than physical activity at baseline.

The program also moved participants towards the recommended ≥7 h of sleep per night for adults [26]. In a recent systematic review of prospective studies, Shan and colleagues demonstrated that individuals who sleep an average of 7–8 h per night have the lowest diabetes risk compared to those who sleep more or less than this [31]. A 7 to 8 h sleep duration has also been associated with lower incidence of hypertension, stroke and coronary heart disease [32]. The combined effects of higher physical activity levels, greater sleep duration, and lower sodium-to-potassium intake ratio may have contributed to the overall reduction in systolic blood pressure observed [28, 30, 32]. The risk of cardiovascular disease rises with increasing blood pressure levels in a continuous manner [33].

Despite the favourable effects on health behaviours, reductions in glucose levels or insulin resistance were not observed, in contrast to our previous study in mothers alone [10]. In comparison to mothers in that study, those in the current study were younger (mean 38 years, SD 5 vs. 40 years, SD 5) with more recent pregnancy (1.9 years ago SD 1.1 vs. 3.5 years ago SD 2.0) and lower postpartum weight retention (1.9 kg SD 5.4 vs. 4.4 kg SD 5.4). Prediabetes (impaired glucose tolerance and/or impaired fasting glucose) was substantially less prevalent (15% vs. 37%). These differences are likely important in terms of ability to demonstrate effects on glucose handling. O’Reilly and colleagues [27] conducted a large diabetes prevention program randomized controlled trial among women within 1 year of a GDM pregnancy (MAGDA-DPP). The intervention included 1 individual session, 5 group sessions, and 2 telephone contacts. The overall prevalence of prediabetes was 12%, similar to our current study. At the 1-year assessment, no between group conclusive differences were observed for fasting glucose, oral glucose tolerance testing values, or blood pressure measures. This raises the possibility that even though postpartum diabetes risk is highest during the first 5 years postpartum [2], prediabetes and insulin resistance may be more apparent beyond 2 years postpartum and thus impact on these measures may be more evident later in life.

The findings in our Canadian study and those of O’Reilly and colleagues in Australia illustrate the challenges of recruitment. The MAGDA investigators enrolled and randomized 7% of potentially eligible participants; we enrolled 6% of potentially eligible participants (Fig. 1). Higher recruitment rates may be achievable in other settings; for example, in an ongoing trial in China (Tianjin trial) [34], 25% of potentially eligible women were enrolled. Insulin resistance was not measured in the trial by O’Reilly and colleagues but in our single arm intervention trial, the baseline HOMA-IR was normal at 1.2 while in the Tianjin trial it was close to 2. There appears to be a need in some settings to develop better strategies to attract at-risk individuals into diabetes prevention programs. Although we did recruit participants from various ethnicities, including those at high risk for diabetes, their socio-economic status was arguably high (e.g., two thirds had completed a university degree). Moreover, baseline cardiometabolic measures were within normal limits.

Our program focused on the couple rather than on the mother alone. To attract couples at risk, however, may require more focused and effective motivational strategies and knowledge sharing directed at both parents during pregnancy, given the variety of barriers to postpartum participation that have been described [35]. An alternative is to build a postpartum diabetes prevention program into the structure of care. This approach was tested through a cluster randomized pragmatic trial conducted among 44 Kaiser Permanente clinics in mothers [36]. However, with such an approach, engagement and attendance is not guaranteed. While 13 telephone coaching sessions were offered to active arm participants in that trial, half did not participate in even one conversation. In the MAGDA trial, 52% received the ‘minimum’ intervention of 1 individualized session and 1 group session; interestingly, greater engagement was achieved in a telephone coaching strategy examined by the MAGDA investigators in a single arm intervention study [37]. In our single arm intervention study, 87% of those enrolled attended at least one session; moreover, more than 60% of fathers and 75% of mothers attended at least 3 of the 5 scheduled sessions. The active nature of the sessions (e.g., cooking, exercising) may have enhanced their attractiveness.

We have demonstrated that it is possible to engage both partners and mothers in a health behavior change program and that both will derive benefit in terms of improvement in these behaviors. The blood pressure lowering observed also signal a cardiometabolic impact of these behavioural changes. There are several limitations to our study. First, it is not a randomized controlled trial but rather a single-arm intervention study; thus there is no control arm with which to compare our findings. However, this design was deliberately chosen as part of a process of iterative refinement. We ultimately seek to develop an intervention that merits the large investment that a randomized controlled trial requires [38]. Importantly, although we have demonstrated engagement and impact on health behaviors, we have also determined that recruitment strategies must be further refined to attract those most at risk and in whom metabolic benefit can be corroborated. Finally, the ideal randomized controlled trial will include not only health behaviour, engagement, and cardiometabolic outcomes, but also clinical outcomes such as recurrent GDM and incident type 2 diabetes.

## Conclusion

In conclusion, our study demonstrates that recruiting couples within 5 years of a GDM pregnancy is no more challenging than the recruitment of mothers alone; better strategies are needed overall. Among both partners and mothers who enrolled, however, attendance was high and health behaviour change was achieved. This is an important step towards the ultimate aim is to leverage a GDM history to stimulate diabetes risk reduction not only in mothers but in partners and the family as a whole.

## Additional file

Additional file 1: **Table S1.** Change in food intakes and eating behaviors. MEQ: Mindful eating questionnaire; Score out of 4; Higher values = more mindfulness WEL: Weight efficacy lifestyle questionnaire; Score out of 9; Higher values = higher self-efficacy. **Table S2.** Change in physical activity behaviors. PASP-Q: Physical activity and sedentary behaviour questionnaire; MVPA: Moderate to vigorous physical activity; OSPAQ: Occupational Sitting And Physical Activity Questionnaire. **Table S3.** Change in cardiometabolic parameters among the MoMM and MoMM-ii mothers. BMI: Body mass index; HOMA-IR: Homeostasis model-assessment-estimated insulin resistance. (DOCX 51 kb)

## Abbreviations

BMI: Body mass index
CI: Confidence interval
DBP: Diastolic blood pressure
GDM: Gestational diabetes
HOMA-IR: Homeostatic Model Assessment Insulin resistance
ISI_0,120_: Insulin sensitivity index
MEQ: Mindful Eating Questionnaire
OSPAQ: Occupational Sitting and Physical Activity Questionnaire
PASP-Q: Physical Activity and Sedentary Behaviour Questionnaire
PSQI: Pittsburgh Sleep Quality Index
SBP: Systolic blood pressure
SD: Standard deviation
WEL: Weight Efficacy Lifestyle
