# Supplementary material for: Health behaviour changes in partners of women with recent gestational diabetes: a phase IIa trial
Source: BMC Public Health. 2018 May 2;18:575. doi: 10.1186/s12889-018-5490-x (PMC5930949; doi:10.1186/s12889-018-5490-x)
Supplement: Supplementary file 1 — Table S1. Change in food intakes and eating behaviors. MEQ: Mindful eating questionnaire; Score out of 4; Higher values = more mindfulness WEL: Weight efficacy lifestyle questionnaire; Score out of 9; Higher values = higher self-efficacy. Table S2. Change in physical activity behaviors. PASP-Q: Physical activity and sedentary behaviour questionnaire; MVPA: Moderate to vigorous physical activity; OSPAQ: Occupational Sitting And Physical Activity Questionnaire. Table S3. Change in cardiometabolic parameters among the MoMM and MoMM-ii mothers. BMI: Body mass index; HOMA-IR: Homeostasis model-assessment-estimated insulin resistance. (DOCX 51 kb) [file 12889_2018_5490_MOESM1_ESM.docx]

**Health behaviour changes in partners of women with recent gestational diabetes: a phase IIa trial**

**Additional file 1**

**Table S1. Change in food intakes and eating behaviors**

|  | All | | Partners | | | Mothers | | |
| --- | --- | --- | --- | --- | --- | --- | --- | --- |
| Variables | Baseline  mean (sd)  or n (%) | Mean change  [95% CI] | n | Baseline  mean (sd)  or n (%) | Mean change [95% CI] | n | Baseline  mean (sd)  or n (%) | Mean change  [95% CI] |
| Energy, kcal/day | 2100 (471) | -82 [-210, 47] | 30 | 2104 (553) | -42 [-212, 128] | 24 | 2095 (356) | -132 [-342, 78] |
| Proteins, % total energy intake | 18.3 (3.1) | 0.1 [-0.9, 1.1] | 30 | 18.1 (2.6) | -0.2 [-1.6, 1.3] | 24 | 18.6 (3.6) | 0.4 [-1.0, 1.9] |
| Carbohydrates, % total energy intake | 43.4 (7.0) | -0.4 [-2.2, 1.3] | 30 | 43.8 (5.8) | 0.2 [-2.0, 2.4] | 24 | 43.1 (8.4) | -1.3 [-4.3, 1.8] |
| Fibers, g per 1,000 kcal | 8.5 (2.1) | 0.7 [0.02, 1.3] | 30 | 8.3 (1.6) | 0.4 [-0.4, 1.3] | 24 | 8.8 (2.6) | 0.9 [-0.1, 2.0] |
| Lipids, % total energy intake | 37.7 (6.6) | 0.3 [-1.1, 1.8] | 30 | 37.0 (5.4) | -0.2 [-1.9, 1.5] | 24 | 38.5 (8.0) | 1.0 [-1.6, 3.6] |
| MUFA, % total energy intake | 15.6 (3.6) | 0.1 [-0.7, 1.0] | 30 | 15.2 (3.1) | 0.0 [-0.01, 0.01] | 24 | 16.2 (4.2) | 0.0 [-1.5, 1.5] |
| PUFA, % total energy intake | 7.1 (2.2) | 0.9 [0.3, 1.5] | 30 | 7.0 (2.3) | 0.006 [-0.002, 0.015] | 24 | 7.2 (2.0) | 1.2 [0.2, 2.2] |
| SFA, % total energy intake | 11.8 (2.7) | 0.5 [-0.5, 1.5] | 30 | 11.6 (2.6) | 0.004 [-0.010, 0.18] | 24 | 12.0 (2.9) | 0.7 [-0.9, 2.3] |
| Sodium, mg/day | 2860 (984) | -285 [-564, -6] | 30 | 2812 (993) | -188 [-551, 174] | 24 | 2912 (991) | -407 [-868, 54] |
| Potassium, mg/day | 2729 (705) | 80 [-140, 320] | 30 | 2702 (797) | 126 [-200, 452] | 24 | 2763 (585) | 45 [-301, 391] |
| Sodium-to-potassium ratio | 1.1 (0.3) | -0.12 [-0.18, -0.05] | 30 | 1.1 (0.3) | -0.09 [-0.19, -0.001] | 24 | 1.1 (0.3) | -0.15 [-0.25, -0.04] |
| Grains products, servings/day | 7.2 (3.1) | -1.1 [-2.0, -0.2] | 30 | 7.4 (3.0) | -0.8 [-2.1, 0.5] | 24 | 6.8 (3.3) | -1.4 [-2.7, -0.2] |
| Vegetables & Fruits, servings/day | 4.6 (2.0) | 0.6 [-0.1, 1.2] | 30 | 4.2 (1.7) | 0.7 [-0.1, 1.4] | 24 | 5.1 (2.2) | 0.5 [-0.7, 1.7] |
| Meat & Alternatives, servings/day | 4.0 (1.6) | 0.0 [-0.4, 0.4] | 30 | 4.0 (1.9) | -0.1 [-0.8, 0.7] | 24 | 3.9 (1.2) | 0.2 [-0.3, 0.6] |
| Milk & Alternatives, servings/day | 1.9 (1.2) | 0.0 [-0.3, 0.3] | 30 | 2.0 (1.3) | 0.1 [-0.4, 0.6] | 24 | 1.9 (1.2) | -0.1 [-0.6, 0.3] |
| MEQ_average | 2.8 (0.3) | 0.2 [0.1, 0.3] | 45 | 2.8 (0.3) | 0.1 [0.0, 0.2] | 45 | 2.8 (0.4) | 0.2 [0.1, 0.3] |
| MEQ_awareness | 2.7 (0.6) | -0.2 [-0.3, 0.0] |  | 2.7 (0.6) | -0.3 [-0.4, -0.1] |  | 2.7 (0.6) | -0.03 [-0.18,0.13] |
| MEQ_distraction | 3.1 (0.6) | -0.1 [-0.2, 0.1] |  | 3.1 (0.5) | -0.2 [-0.4, -0.1] |  | 3.0 (0.6) | 0.1 [-0.1, 0.3] |
| MEQ_dishinibition | 2.8 (0.6) | 0.7 [0.6, 0.8] |  | 2.7 (0.7) | 0.8 [0.6, 1.0] |  | 2.9 (0.6) | 0.6 [0.5, 0.8] |
| MEQ_emotional | 3.3 (0.6) | -1.2 [-1.4, -1.0] |  | 3.4 (0.5) | -1.4 [-1.7, -1.1] |  | 3.1 (0.7) | -1.0 [-1.3, -0.7] |
| MEQ_external cues | 2.3 (0.5) | 1.6 [1.4, 1.9] |  | 2.2 (0.5) | 1.7 [1.4, 2.1] |  | 2.4 (0.6) | 1.5 [1.1, 1.9] |
| WEL average | 6.4 (1.5) | 0.1[-0.1, 0.3] | 45 | 6.6 (1.5) | 0.1 [-0.3, 0.4] | 45 | 6.3 (1.6) | 0.2 [-0.1, 0.4] |

MEQ: Mindful eating questionnaire; Score out of 4; Higher values = more mindfulness

WEL: Weight efficacy lifestyle questionnaire; Score out of 9; Higher values= higher self-efficacy

**Table S2. Change in physical activity behaviors**

|  |  | All | | Partners | | | Mothers | | |
| --- | --- | --- | --- | --- | --- | --- | --- | --- | --- |
| Variables | unit | Baseline mean (sd) | Mean change  [95% CI] | n | Baseline mean (sd) | Mean change  [95% CI] | n | Baseline mean (sd) | Mean change  [95% CI] |
| **Objectives measures** |  |  |  |  |  |  |  |  |  |
| Steps | counts/d | 7481 (2314) | 1355 [740, 1970] | 44 | 7553 (2871) | 1645 [730, 2561] | 44 | 7410 (1606) | 1065 [215, 1915] |
| Sedentary behaviors | % wear time | 67.9 (7.7) | -1.21 [-2.69, 0.003] | 35 | 70.0 (0.1) | -0.02 [-0,04, 0.01] | 36 | 65.9 (7.3) | -0.01 [-0.03, 0.01] |
| MVPA | min/wk | 94.5 (98.5) | 27.0 [5.1, 49.0] | 35 | 105.0 (114.8) | 36.4 [1.4, 71.4] | 36 | 85.4 (79.8) | 18.0 [-10.2, 46.2] |
| **Self-reported measures** |  |  |  |  |  |  |  |  |  |
| PASP-Q MVPA | min/wk | 79.8 (115.6) | 31.4 [-3.6, 66.4] | 45 | 100.7 (123.4) | 33.5 [-24.1, 91.2] | 46 | 59.5 (104.8) | 29.3 [-13.3, 71.8] |
| PASP-Q Walk | hours/wk | 5.1 (6.0) | 2.0 [0.7, 3.2] | 45 | 4.5 (4.1) | 2.1 [0.9, 3.4] | 46 | 5.7 (7.5) | 1.8 [-0.4, 4.0] |
| PASP-Q Muscle strengthening | sessions/ wk | 0.6 (1.3) | 0.4 [0.1, 0.7] | 45 | 0.7 (1.2) | 0.4 [0.0, 0.7] | 46 | 0.5 (1.3) | 0.4 [0.0, 0.9] |
| PASP-Q Overall sitting | hours/d | 7.6 (3.3) | -0.8 [-1.4, -0.3] | 45 | 8.5 (3.2) | -1.0 [-1.8, -0.2] | 46 | 6.8 (3.0) | -0.7 [-1.5, 0.1] |
| OSPAQ Sitting at work | hours/d | 5.6 (3.0) | -0.5 [-1.1, 0.2] | 38 | 5.8 (3.1) | -0.6 [-1.4, 0.2] | 23 | 5.2 (2.7) | -0.2 [-1.3, 0.8] |
| OSPAQ Standing at work | hours/d | 1.2 (1.6) | 0.03 [-0.24, 0.31] | 38 | 1.2 (1.6) | 0.1 [-0.3, 0.4] | 23 | 1.1 (1.6) | -0.03 [-0.50, 0.44] |

PASP-Q Physical activity and sedentary behaviour questionnaire

MVPA: Moderate to vigorous physical activity

OSPAQ: Occupational Sitting And Physical Activity Questionnaire

**Table S3 Change in cardiometabolic parameters among the MoMM and MoMM-ii mothers combined**

| **Variable** | **n** | **Baseline**  **Mean** | **SD** | **Post-program Mean** | **SD** | **Mean change** | **95% CI** | |
| --- | --- | --- | --- | --- | --- | --- | --- | --- |
| Systolic blood pressure, mmHg | 74 | 116.9 | 11.9 | 113.2 | 10.9 | -3.7 | -5.6 | -1.8 |
| Diastolic blood pressure, mmHg | 74 | 70.4 | 8.0 | 68.5 | 8.6 | -1.9 | -3.4 | -0.4 |
| Weight, kg | 74 | 72.4 | 14.7 | 72.3 | 14.8 | -0.1 | -0.5 | 0.3 |
| BMI, kg/m^2^ | 74 | 27.4 | 5.6 | 27.4 | 5.6 | -0.03 | -0.20 | 0.14 |
| Steps, counts/day | 69 | 7253 | 2227 | 8314 | 2880 | 1061 | 501 | 1621 |
| Fasting plasma glucose, mmol/L | 72 | 5.4 | 0.7 | 5.3 | 0.7 | -0.08 | -0.21 | 0.05 |
| 2h plasma glucose, mmol/L | 69 | 6.5 | 2.5 | 6.2 | 2.1 | -0.2 | -0.6 | 0.2 |
| Insulin, μU/mL | 72 | 6.2 | 4.0 | 6.6 | 4.1 | 0.4 | -0.2 | 1.0 |
| 2h-Insulin, μU/mL | 69 | 45.8 | 51.8 | 42.5 | 41.3 | -3.3 | -14.6 | 8.1 |
| HOMA-IR | 72 | 1.5 | 1.1 | 1.6 | 1.1 | 0.1 | -0.1 | 0.2 |

BMI: Body mass index; HOMA-IR: Homeostasis model-assessment-estimated insulin resistance
